# Propensity-Score Matching Analysis for Laser Hemorrhoidoplasty Versus Circumferential Stapler Hemorrhoidectomy: One-Year Outcomes

**DOI:** 10.7759/cureus.71477

**Published:** 2024-10-14

**Authors:** Tran V Hung, Duong V Hai

**Affiliations:** 1 General Surgery, Pham Ngoc Thach University of Medicine, Ho Chi Minh City, VNM; 2 General Surgery, Binh Dan Hospital, Ho Chi Minh City, VNM; 3 General Surgery, University Medical Center, Ho Chi Minh City, VNM

**Keywords:** circumferential stapler hemorrhoidectomy, hemorrhoidal disease, laser hemorrhoidoplasty, laser-therapy, longo operation

## Abstract

Background: Laser hemorrhoidectomy (LHP) is a minimally invasive procedure with less pain, short operative time and length of stay, and a low recurrent rate. This study aimed to analyze the surgical outcomes of the circumferential stapler hemorrhoidectomy (CSH, Longo operation) by propensity score-matching analysis, including perioperative outcomes and quality of life.

Materials and methods: Between March 2022 and March 2023, 216 patients underwent CSH and 198 LHP in Binh Dan Hospital, Ho Chi Minh City, Vietnam. Potential confounding factors for operative outcomes were adjusted by propensity score-matching analysis. The gender, age, Goligher classification, symptoms, Hemorrhoidal Disease Symptom Score (HDSS), and the number of hemorrhoidal columns were matching variables. After 1:1 propensity score-matching, 115 patients from each group were evaluated for perioperative outcomes and compared for a prospective study.

Results: There was no difference in potential preoperative confounders such as gender, hemorrhoid classification, symptoms, and HDSS between the two groups after propensity score-matching. However, there was a difference in age (52 in the Longo group and 43 in the LHP group) and the number of columns (the LHP group had more). Postoperative outcomes such as operative time, blood loss, general complications, and postoperative interventions were less in the LHP group. However, Visual Analog Scale (VAS) (4 vs. 4), length of stay (1 day vs. 1 day), quality of life (both groups improved quality of life after the procedure), and recurrence rate (2 in the Longo group vs. 0 in the LHP group, p=0.5) had no difference between the two groups.

Conclusions: Propensity-score matching analysis showed that the LHP procedure was superior to the Longo operation (CSH) in operative time, blood loss, general complications, and postoperative intervention. Other outcomes such as VAS, length of stay, quality of life, and recurrence rate have no difference.

## Introduction

Gallo et al. found that symptomatic hemorrhoidal disease is a common disorder of the anorectal region, even in developed countries [1]. The pathophysiology of the hemorrhoid is controversial with various theories. The two most important factors are sliding anal cushions by Thomson [2] and vascular abnormality by Lohsiriwat and Aigner et al. [3-5]. Surgical treatment is only for patients not responding to conservative procedures. Simillis et al. stated that the most effective method, excisional hemorrhoidectomy, causes the most pain to the patient [6]. Patients are often hesitant to undergo operations because of pain fear and just a benign condition. So, colorectal surgeons are pursuing operations to treat hemorrhoids without or with less pain. Porrett et al. determined the symptoms of hemorrhoids are very uncomfortable [7] and have little relationship with the grade of the disease; hemorrhoids grade II and III with minimal mucosal prolapse can cause severe symptoms like hemorrhoid grade IV.

The Longo operation (circumferential stapled hemorroidopexy, CSH) shows positive results: short operation time, less postoperative pain, and good surgical results compared to previous methods. Recently, Danys et al. and Weyand et al. showed that laser hemorrhoidoplasty (LHP), using thermal energy by diode laser to treat internal symptomatic hemorrhoids, has become technically simple, minimally invasive, safe, and effective [8,9]. Recently, Poskus et al. and Wee et al. compared LHP with sutured mucopexy and excisional hemorrhoidoplasty [10,11]. Theoretically, although Longo operation and LHP are minimally invasive procedures, it is unknown which will result in better outcomes.

In 2020, we conducted a study comparing LHP and CSH in early and mid-term [12]. The results showed that LHP was superior to CSH in the post-operative pain score, the overall complication rate, the anal stenosis rate, and the length of stay in hospital. However, we compared two treatments in observational cohorts in which randomization could not be performed. As a result, the outcomes could be attributed to the group confounders rather than the treatment effect. Propensity score matching (PSM) is a statistical method to remove the confounding bias from observational cohorts where randomization is impossible.

Consequently, this study compares the one-year outcomes of these two operations for hemorrhoids in terms of effectiveness and quality of life by propensity-score matching.

## Materials and methods

Study design

This is a non-randomized, single-center prospective study. We compared two randomly selected patient groups and used propensity score matching to remove the confounding bias from a non-randomized study. The patients chose the operation for themselves. The sample size was calculated using the Feinstein formula [13]. In a previous study by one of the authors (Duong Van Hai), the surgical results of the two groups of patients were compared with symptomatic hemorrhoids who had undergone the Longo operation and the Milligan-Morgan procedure [14]. The overall complication rate is 20.5%, taking the round number as 20%. When applying the LHP procedure in Vietnam, we expect the complication rate to drop to 10%. The formula for calculating the sample size, according to Feinstein (two-group comparison with a known estimate of treatment effect), is:

2 {P1(100 - P1) + P2(100 - P2)}

P1 is the complication rate of the CSH group (20%). P2 is the desired complication rate of the LHP (which is expected to drop to 10%). So, P2=10%. According to the Feinstein formula, with P1=20% and P2=10%, we need at least 196 patients for each group.

Inclusion Criteria

The inclusion basis was as follows: 1) ≥16 years old; 2) good physical condition (ASA = 1 or II); 3) symptomatic hemorrhoids with degree 2 (not respond to medical treatment) or higher (Goligher classification); 4) the patient agrees to participate in the study.

Exclusion Criteria

The exclusion basis was as follows: 1) inflammatory bowel disease; 2) history of hemorrhoid operation; 3) anal canal stenosis; 4) comorbidity: local (anal fissure, anal fistula) or systemic (cirrhosis, renal failure); 5) anticoagulant (relatively); 6) irritable bowel syndrome with severe diarrhea or constipation.

Operative technique

All patients were under spinal general anesthesia. Prophylaxis antibiotic was given by second-generation cephalosporin 30 minutes before the operation. Only one expert surgeon performed LHP, and many others performed CSH. All patients were in lithotomy and had anoscopy using a 23-mm-diameter proctoscope to examine the whole anal canal.

In laser hemorrhoidoplasty, a semiconductor laser generator with a 1470 nm wavelength, and 13W laser power (Biolitec, Jena, Germany) was used. Suture ligation (degree 2) or mucopexy ligation (degree 3 or 4) was performed. The laser fiber was inserted into the submucosa, advanced to the hemorrhoid cushion, and activated. The procedure may be performed for many piles. Cooling with a cool pack was applied intra-operative. Additional resection of skin tags was performed if necessary.

In the Longo operation, the Covidien EEA (End-to-End Anastomosis) (Medtronic, Minneapolis, MN) stapler was used. The purse-string suture with polypropylene 3-0 was made at least 2 cm above the dentate line. The circular stapler is fully opened, inserted into the purse-string suture, and tied tightly. In female patients, the posterior vaginal wall should be checked to avoid getting stuck in the stitches. Then, the stapler was tightened and held for about 30 seconds to stop bleeding. The sutures were checked, and additional hemostatic sutures were added if bleeding.

Postoperative care

The pain was assessed on days 1, 7, and 14 using the VAS scoring system. Intraoperative events and postoperative complications were estimated. 

Patients were discharged the day after the operation when the pain level was less than four and with no complications. Patients were followed for at least 12 months after the operation (at two weeks, one month, three months, and one year). Patient symptoms and recurrence of hemorrhoids were evaluated at each follow-up visit.

The Hemorrhoidal Disease Symptom Score (HDSS) evaluated the quality of life before and after the procedure for 90 days. HDSS measures the patient-reported frequency of pain, itching, bleeding, soiling, and prolapse. Each symptom is graded 0-4 (0 = never, 1 = less than once a month, 2 = less than once a week, 3 = 1-6 days per week, 4 = every day), giving a total score from 0 to 20 [15]. 

The institutional review board and the ethics committee in biomedical studies of the University of Medicine and Pharmacy at Ho Chi Minh City approved the study (IRB-VN01002/IRB00010293/FWA000223448). All the patients gave written informed consent.

Study population

Between March 2022 and March 2023, 216 patients underwent CSH, and 198 were allocated LHP. The gender, age, Goligher classification, symptoms, HDSS, and the number of columns matched variables. The propensity-score matching analysis was adapted to correct for selection bias. After 1:1 propensity score-matching, 115 patients for each group were selected.

Statistical analysis

A 1:1 matched analysis using nearest-neighbor matching with a caliper distance of 0.2 without replacement was performed based on the estimated propensity score of each patient. Categorical variables were compared with the chi-squared or Fisher's exact test. Continuous variables with symmetrical distribution were compared using the independent t-test. Continuous data with asymmetrical distribution were compared using the Mann-Whitney U test. Data were presented as mean ± SD and n (%) when appropriate, and p < .05 was considered statistically significant. Data were analyzed using IBM SPSS Statistics for Windows, version 26 (IBM Corp., Armonk, NY) and R version 4.4 (R Foundation for Statistical Computing, Vienna, Austria, https://www.R-project.org/).

## Results

Patient characteristics

Before matching, there were significant differences in Goligher classification, HDSS, and the number of columns. However, after matching, there were significant differences in age (the Longo group was older than the LHP group, 52 vs. 43) and the number of columns (the LHP group had more hemorrhoid columns than the Longo group) (Table 1).

**Table 1:** Patient characteristics according to operation type before and after matching. P<0.05 was considered statistically significant. LHP: laser hemorrhoidectomy; HDSS: Hemorrhoidal Disease Symptoms Score measures the patient-reported frequency of pain, itching, bleeding, soiling, and prolapse. Each symptom is graded 0-4 (0 = never, 1 = less than once a month, 2 = less than once a week, 3 = 1-6 days per week, 4 = every day), giving a total score from 0 to 20. * results of t-test; ** results of chi-square test.

Variables		Before matching			After matching		
Procedure		Longo (n=216)	LHP (n=198)	P	Longo (n=115)	LHP (n=115)	P
Gender	Male	132 (61.1%)	120 (60.6%)	0.91*	59 (51.3%)	59 (51.3%)	1*
	Female	84 (38.9%)	78 (39.4%)		56 (48.7%)	56 (48.7%)	
Age		47±14.1	45.8±13.5	0.1*	50.9±1.4	45.5±1.2	0.004*
Classification of hemorrhoid S	Grade 2	6 (2.8%)	18 (9.1%)	0.001**	6 (5.2%)	7 (6.1%)	0.6**
	Grade 3	130 (60.2%)	33 (16.7%)		30 (26.1%)	33 (28.7%)	
	Grade 4	5 (2.3%)	1 (0.5%)		4 (3.9%)	1 (0.9%)	
	Miscellaneous	75 (34.7%)	146 (73.7%)		75 (65.2%)	74 (64.3%)	
Symptoms	Bleeding	127(58.8%)	125 (63.1%)	0.2**	61 (53%)	70 (60.9%)	0.5**
	Prolapse	67 (31%)	48 (24.2%)		40 (34.8%)	31 (27%)	
	Pain	18 (8.3%)	16 (8.1%)		11 (9.6%)	9 (7.8%)	
	Soiling	4 (1.9%)	9 (4.5%)		3 (2.61%)	5 (4.35%)	
HDSS		9.5±3.2	9.4±3.4	0.6*	9.8±3.8	9±3.1	0.36*
Number of hemorrhoid columns	1	6 (2.8%)	7 (3.5%)	0..000*	5 (4.3%)	4 (3.5%)	0.001**
	2	19 (8.8%)	8 (4%)		11 (9.6%)	4 (3.5%)	
	3	187 (86.6%)	88 (44.4%)		97 (84.3%)	53 (46.1%)	
	4	4 (1.9%)	76 (38.4%)		2 (1.74%)	42 (36.5%)	
	5	0	18 (9.1%)		0	11 (9.6%)	
	6	0	1 (0.5%)		0	1 (0.9%)	

Comparison of the operative outcomes

Before matching, the LHP group was superior to the Longo group in operative time, blood loss, complications, postoperative interventions, length of stay, and recurrence rate. The Longo group was superior to the LHP group in complementary procedures and VAS day 1. However, after matching, the LHP group is superior to Longo only in operative time, blood loss, complications, and postoperative interventions. Other variables (complementary procedures, VAS day 1, length of stay, and recurrence rate) had no significant differences in the two groups (Table 2).

**Table 2:** Intra- and postoperative variables before and after matching. P<0.05 was considered statistically significant. LHP: Laser hemorrhoidectomy; VAS: Visual Analog Scale. * results of t-test; ** results of chi-square test.

Variables		Before matching			After matching		
Procedure		Longo (n=216)	LHP (n=198)	P	Longo (n=115)	LHP (n=115)	P
Operative time (minute)		36.9 ±11.2	29.4±9.7	0.000*	37.3±1.04	29.2±0.9	0.001*
Blood loss (ml)		13.4±12.1	9.7±8.8	0.000*	12.1±0.9	9.3±0.8	0.004*
Additional procedure	Yes (excision of external hemorrhoids, skin tags, anal polyp)	75 (34.7%)	146 (73.7%)	0.001**	75 (65.2%)	74 (64.3%)	0.9**
	No	141 (65.3%)	52 (26.3%)		40 (34.8%)	41 (35.7%)	
	VAS	Day 1	4.2±1.5		5±1.9	0.000*		4.9±0.1	4.7±0.2	0.1*
Day 7		0.56±0.69	0.66±0.64	0.14*	0.6±0.06	0.6±0.06	0.5*
Day 14		0.56±0.69	0.65±0.63	0.17*	0.6±0.06	0.6±0.06	0.6*
Complications	Bleeding	5 (2.3%)	5 (2.5%)	0.01**	3 (2.6%)	4 (3.5%)	0.01**
	Urinary retention	17 (7.9%)	14 (7.1%)		11 (9.6%)	8 (7%)	
	Anal stricture	11 (5.1%)	0		8 (7%)	0	
Postoperative interventions	Gauze/balloon compression	4 (1.8%)	5 (2.5%)	0.04**	2 (1.7%)	4 (3.5%)	0.04**
	Hemostatic suture	1 (0.5%)	0		1 (0.9%)	0	
	Urinary catheterization	17 (7.9%)	14 (7.1%)		11 (9.6%)	8 (7%)	
	Anal dilatation	6 (2.8%)	0		4 (3.5%)	0	
	Anoplasty	5 (2.3%)	0		4 (3.5%)	0	
Length of stay (day)		1±0.9	1±0.2	0.03*	1.01±0.01	1.03±0.02	0.18*
Follow-up (week)		52	52	1*	52	52	1*
Recurrence rate		7 (3.2%)	0	0.01*	2 (1.7%)	0	0.5*
Recurrence time	3 months	6 (85.7%)	0	0.01*	2 (1.7%)	0	0.5*
	5 months	1 (14.3%)	0		0	0	
Recurrence symptoms	Prolapse	7 (3.2%)	0	0.01*	2 (1.7%)	0	0.5*
Recurrence treatment	Medications	4 (57.1%)	0	0.01*	0	0	0.5*
	Hemorrhoidectomy	3 (42.9%)	0		3 (2.6%)	0	

## Discussion

Longo operation (CSH) is a long-developed technique with advantages such as less operative time, less postoperative pain, and fewer postoperative complications [16,17]. LHP is a new minimally invasive procedure and has been reported to have advantages in intraoperative and postoperative [18-20].

This is the first study to use propensity-score matching to compare the surgical outcomes of the LHP and Longo procedures. The results showed that LHP is superior to the Longo procedure regarding operative time (29.2 vs. 37.3 min), blood loss (9.3 vs. 12.1 ml), complications (10.5% vs. 19.2%), and postoperative interventions (10.5% vs. 19.2%). Other critical variables in VAS day one (4.9 vs. 4.7), length of stay (1.01 vs. 1.03 day), and recurrence rate (0 vs.1.7%, p=0,5) have no significant differences between the two groups. These are the most important findings of this study (Table 2).

The average operation time is shorter in the LHP group. Many studies worldwide have shown that the performing time of the LHP technique varies from 10 to 33 minutes [18,19]. This is possible due to the operating speed, whether or not additional techniques are performed, the number of hemorrhoidal columns, and classification. In this study, these factors did not affect the operative time (Tables 1, 2).

The intraoperative blood loss is higher in the Longo group (12.1 vs. 9.3 ml in the LHP group, p=0,004). Postoperative bleeding was recorded in both groups, 3 in the Longo group and 4 in the LHP group, which were not significantly different. One case in the Longo group required surgical intervention for bleeding control. Other patients of the two groups were treated conservatively with hemostatic drugs, gauze/balloon compression in the anal canal. No blood transfusion is needed. Bleeding is a common complication in the Longo procedure (0.18% to 33%) [21, 22] and LHP group (0.6 to 10%) [23]. In the LHP group, we have two cases (1.8%) of late bleeding (after two weeks) when reusing anti-platelet aggregation drugs for cardiovascular diseases. Maloku et al. also recorded a case of bleeding from a patient taking aspirin [18].

Postoperative pain is the most critical complication that causes patients to be uncomfortable and not want surgery. In LHP, the anal canal mucosa is preserved, and no tissue excision is performed, so no pain or less pain results. However, in this study, the VAS is comparative. Recent studies (not using the PSM technique) showed that LHP caused less pain than other procedures [19]. VAS does not depend on additional intervention (Table 2).

The anal stenosis is higher in the Longo group (eight cases, 7%) compared to LHP (no case), with a significant difference (p=0.01). The anal stenosis is critical after hemorrhoidal surgery due to its influence on the patient’s life. The stenosis risk factors in the Longo procedure include higher grade in classification, increased sphincter tone, and sphincter injuries [21,24,25]. In our study, four cases are successfully managed by anal dilatation; only one case has to undergo anoplasty as a secondary intervention (Table 2). 

The complication rate was higher in the Longo group (19.2%) than in the LHP group (10.5%), with a significant difference (p=0.01). The complication rate of the Longo procedure was approximately the same as in our previous study, 15% [14].

HDSS evaluates the quality of life before the procedure and after the operation for 90 days. HDSS is comparable in two groups. The quality of life is clearly improved after the operation, with a significant difference (Table 2). A recent study showed that hemorrhoidal disease negatively affects the quality of life, according to health-related quality of life (HRQoL). Treating hemorrhoidal disease clearly increases the quality of life [26]. Both procedures improved the HDSS; however, the success rate and complications differ.

The recurrence rate is higher in the Longo group (two cases, 1.7%) but not statistically significant in the LHP group (p=0.5) in one-year follow-up. The median recurrence rate of the Longo procedure is 6.9% [24]. Risk factors are grade IV classification and technical characteristics of the Longo procedure [17]. The recurrence rate of LHP of LHP procedure varies at different follow-up times: 8.8% in six months and 34% in five years [27]. It is clear that the recurrence rate increases as the follow-up time increases.

This study has some limitations. There is no agreed protocol for LHP operations, so the outcomes may differ from the techniques used in each study. The Longo procedure and LHP indications were selected according to the patient’s preference. Further, only one surgeon was in the LHP group, while many were in the Longo group. Although propensity score matching is used, some biases cannot be eliminated (such as selection and chance). This is a single-center study, so it has limitations when translated to a broader population of patients. Randomized controlled, multicenter studies, larger sample sizes, and longer follow-ups are needed to resolve these limitations.

## Conclusions

In this study, using propensity-score matching analysis, we demonstrated that the LHP procedure is superior to the Longo procedure in terms of operative time, intraoperative blood loss, complication rate, and postoperative interventions. However, the LHP procedure is the same as the Longo procedure regarding postoperative pain (according to VAS), length of stay (day), and recurrence rate. So, the LHP is a safe, effective, minimally invasive procedure. These findings showed that the LHP is one option for symptomatic hemorrhoids in clinical practice.

The quality of life has clearly improved after both operations, with a significant difference.
